# Auranofin-mediated inhibition of PI3K/AKT/mTOR axis and anticancer activity in non-small cell lung cancer cells

**DOI:** 10.18632/oncotarget.6516

**Published:** 2015-12-09

**Authors:** Hongyu Li, Jing Hu, Shuhong Wu, Li Wang, Xiaobo Cao, Xiaoshan Zhang, Bingbing Dai, Mengru Cao, Ruping Shao, Ran Zhang, Mourad Majidi, Lin Ji, John V. Heymach, Michael Wang, Shiyang Pan, John Minna, Reza J. Mehran, Stephen G. Swisher, Jack A. Roth, Bingliang Fang

**Affiliations:** ^1^ Department of Thoracic and Cardiovascular Surgery, University of Texas MD Anderson Cancer Center, Houston, Texas, USA; ^2^ Department of Thoracic/Head & Neck Medical Oncology, University of Texas MD Anderson Cancer Center, Houston, Texas, USA; ^3^ Department of Lymphoma, University of Texas MD Anderson Cancer Center, Houston, Texas, USA; ^4^ Jilin Province Cancer Hospital, Changchun, Jilin, China; ^5^ Department of Laboratory Medicine, The First Affiliated Hospital of Nanjing Medical University, Nanjing, Jiangsu, China; ^6^ Hamon Center for Therapeutic Oncology, The Harold C. Simmons Comprehensive Cancer Center, University of Texas Southwestern Medical Center, Dallas, Texas, USA

**Keywords:** lung cancer, drug repurposing, anticancer agent, biomarkers

## Abstract

Auranofin, a gold complex that has been used to treat rheumatoid arthritis in clinics and has documented pharmacokinetic and safety profiles in humans, has recently been investigated for its anticancer activity in leukemia and some solid cancers. However, auranofin's single agent activity in lung cancer is not well characterized. To determine whether auranofin has single agent activity in lung cancer, we evaluated auranofin's activity in a panel of 10 non-small cell lung cancer (NSCLC) cell lines. Cell viability analysis revealed that auranofin induced growth inhibition in a subset of NSCLC cell lines with a half maximal inhibitory concentration (IC_50_) below 1.0 μM. Treatment with auranofin elicited apoptosis and necroptosis in auranofin-sensitive cell lines. Moreover, the susceptibility of NSCLC cells to auranofin was inversely correlated with TXNRD1 expression in the cells. Transient transfection of the TXNRD1-expressing plasmid in auranofin-sensitive Calu3 cells resulted in partial resistance, indicating that high TXNRD level is one of causal factors for resistance to auranofin. Further mechanistic characterization with proteomic analysis revealed that auranofin inhibits expression and/or phosphorylation of multiple key nodes in the PI3K/AKT/mTOR pathway, including S6, 4EBP1, Rictor, p70S6K, mTOR, TSC2, AKT and GSK3. Ectopic expression of TXNRD1 partially reversed auranofin-mediated PI3K/AKT/mTOR inhibition, suggesting that TXNRD1 may participate in the regulation of PI3K/AKT/mTOR pathway. Administration of auranofin to mice with xenograft tumors derived from NSCLC cells significantly suppressed tumor growth without inducing obvious toxic effects. Our results demonstrated feasibility of repurposing auranofin for treatment of lung cancer.

## INTRODUCTION

Lung cancer is one of the top 2 leading causes of death in the United States and other developed countries [1]. Globally, lung cancer's annual incidence is approximately 1.8 million, with an annual mortality of approximately 1.6 million [2]. The 5-year overall survival rate for lung cancer patients has improved only moderately over the past 4 decades despite the use of many therapeutic modalities [3]. Therefore, development of new therapeutic strategies for this deadly disease is urgently needed. However, anticancer drug development is impeded by high failure rates, primarily because of lack of efficacy, inability to identify responders, and intolerable toxic effects [4]. Safety concerns are one of the major causes of discontinuation of drug development preclinically and clinically and discontinuation of a drug after FDA approval [5, 6]. Thus, substantial efforts have been devoted to repurposing FDA-approved drugs with known safety in humans for new indications [7–10]. Indeed, thalidomide has been successfully repurposed for the treatment of multiple myeloma [7]. Our recent finding that ibrutinib, a drug approved for the treatment of B cell malignancy, has effective anti-EGFR activity in erlotinib-resistant lung cancer [9] has quickly translated to a clinical trial, demonstrating that drug repurposing can have a rapid impact on cancer therapy.

Using a genome-wide synthetic lethality screen of a small interfering RNA (siRNA) library, we recently found that thioredoxin reductases 1 (TXNRD1) is a synthetic lethal partner of AKT in non-small cell lung cancer (NSCLC) cells [11]. Inhibiting TXNRD activity with siRNA or the small-molecule inhibitor auranofin dramatically sensitized NSCLC cells to treatment with the AKT inhibitor MK2206 [11]. Auranofin (Ridaura) is a gold complex that has been used by physicians to treat rheumatoid arthritis since the 1980s [12], and it has documented pharmacokinetic and safety profiles in humans [13–15]. Long-term use of auranofin is well tolerated in both juvenile and elderly patients [13–15]. The common side effects are loose stools or diarrhea, skin rash, stomatitis or conjunctivitis, and proteinuria [14, 16]. The dropout rate due to adverse events in the treatment group was lower than in the placebo group in clinical trials [15], demonstrating that auranofin has an excellent safety profile. Recent studies have demonstrated that auranofin is highly effective against *Entamoeba histolytica* infection, leading to a quick FDA approval for the treatment of amebiasis with auranofin [17]. The use of auranofin to treat various cancers has also been explored [18–20], and auranofin is currently in clinical trials for the treatment of leukemia [21]. A recent study on the effects of auranofin in chronic lymphocytic leukemia revealed that auranofin overcame apoptosis resistance mediated by protective stromal cells [22], suggesting that auranofin may target the tumor microenvironment as well. Moreover, patients with rheumatoid arthritis treated with gold had lower malignancy rates than those not treated with gold [23], further supporting the feasibility of using auranofin for cancer therapy. To further explore the possibility of using auranofin for treatment of lung cancer, we determined single agent activity of auranofin in a panel of lung cancer cell lines. Here we report auranofin's anticancer activity in non-small cell lung cancer cell lines *in vitro* and vivo. Our results revealed that auranofin inhibit PI3K/AKT/mTOR axis and induce potent anticancer activity in a subset of lung cancer cell lines.

## RESULTS

### Auranofin-mediated anti-lung cancer activity *in vitro*

**Figure 1:**
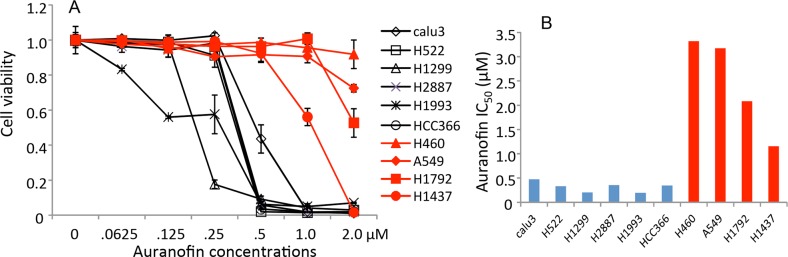
*In vitro* activity of auranofin in NSCLC cells **A.** Dose-response of auranofin in 10 NSCLC cell lines determined by cell viability assays. The values in control cells were set as 1. Data are presented as mean ± standard deviation (SD) of a quadruplet assay. **B.** Auranofin IC_50_ values (μM) determined by cell viability assays.

Our recent study revealed that TXNRD1 siRNA and its inhibitor auranofin were synthetically lethal with the novel AKT inhibitor MK2206 in lung cancer cells and dramatically enhanced the efficacy of MK2206 in both *in vitro* and *in vivo* models [11]. To further investigate the potential application of auranofin for lung cancer therapy, we determined the single-agent activity of auranofin in 10 NSCLC cell lines. The cells were treated with different concentrations of auranofin ranging from 62.5 nM to 2μM. Dose-dependent cell viability was determined using the sulforhodamine B assay, as described previously [9, 24]. Results showed that NSCLC cells had differential sensitivity to auranofin (Figure 1). Six of the 10 cell lines tested had a half maximal inhibitory concentration (IC_50_) below 1.0 μM and 3 cell lines had an IC_50_ above 2 μM, the highest concentration tested. H1437 had intermediate sensitivity (IC_50_ = 1.1μM). This result strongly suggested that auranofin may have single agent activity in some NSCLC cells.

### Auranofin induces robust cell death in sensitive lung cancer cells

**Figure 2:**
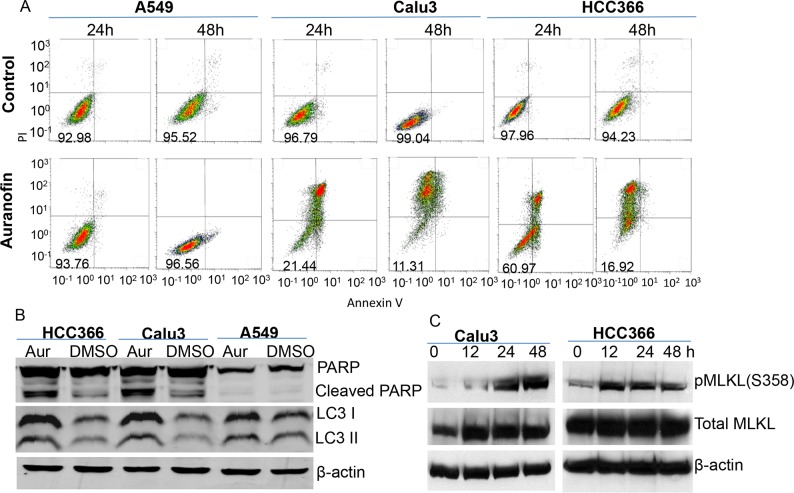
Auranofin-induced cytotoxicity in NSCLC cells **A.** Auranofin-sensitive Calu3 and HCC366 cells and auranofin-resistant A549 cells were treated with 0.5μM auranofin or DMSO for 24 or 48 h. Cell death was determined by fluorescence-activated cell sorting after staining with annexin V and propidium iodide. The numbers in the lower left box of each graph represent the number of surviving cells. **B.** Western blot analysis of PARP and LC3 levels after treatment with 0.5μM auranofin for 24 hours. β-actin is used as a loading control. **C.** Western blot analysis on phosphorylation of MLKL in Calu3 and HCC366 after treatment with 0.5μM auranofin for different time as indicated. β-actin is used as a loading control.

We sought to determine whether auranofin triggers cytostatic or cytotoxic effects in lung cancer cells. Lung cancer cell lines Calu3, HCC366, and A549 were treated with 0.5μM auranofin for 12-48 hours. Cells treated with DMSO served as a control. Apoptosis was measured by fluorescence-activated cell sorting after staining with annexin V and propidium iodide. Results showed that treatment with 0.5μM auranofin for 24-48 hours induced robust cell death or apoptosis in Calu3 and HCC366 cells. Only background cell death was detected in A549 cells at all time points tested, whereas in Calu3 and HCC366 cells, background cell death was detected at the early time point (12 hours) and robust cell death was detected thereafter (Figure 2A). Western blot analysis of cell lysates harvested at 24 hours also demonstrated a dramatic increase in cleaved PARP levels in HCC366 and Calu3 cells, but not in A549 cells (Figure 2B), suggesting that apoptosis is one of the mechanisms of auranofin-induced cell death in auranofin-sensitive lung cancer cells. LC3 II was mildly increased in HCC366 and Calu3 cells after treatment with 0.5μM auranofin for 24 hours, suggesting the presence of some autophagy in auranofin-treated cells. We also investigated whether programmed necrotic cell death or necroptosis [25] is involved in auranofin induced cell killing by Western blot analysis on phosphorylation of mixed lineage kinase domain-like protein (MLKL), a hallmark of necroptosis [25, 26]. The result showed that treatment of HCC366 and Calu3 cells with 0.5μM auranofin induced time-dependent elevation of phospho-MLKL (Figure 2C). These results demonstrated that auranofin-induced cytotoxicity may be caused by multiple programmed cell death mechanisms.

### TXNRD1 expression is inversely associated with auranofin-mediated anti-lung cancer activity

**Figure 3:**
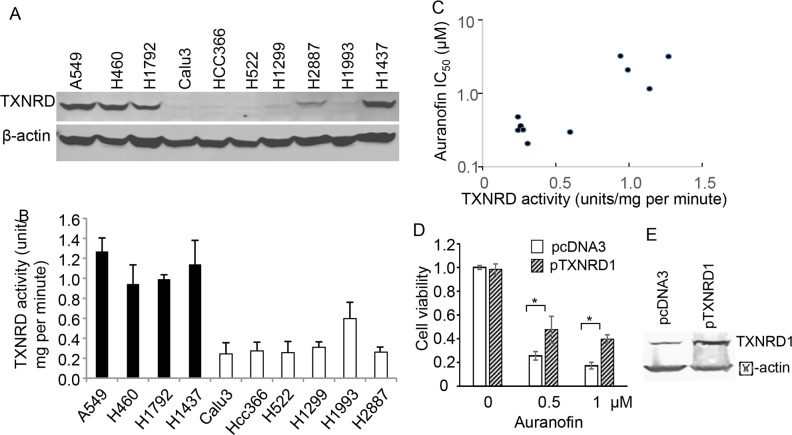
Association of TXNRD1 expression and auranofin's anticancer activity in NSCLC cells **A.** Western blot analysis of TXNRD1 expression in NSCLC cell lines. β-actin is used as a loading control. **B.** TXNRD enzymatic activity levels in NSCLC cell lines. The values represent mean + SD of 3 triplicate assays. **C.** Correlations between TXNRD enzymatic activity and auranofin IC_50_ values in 10 NSCLC cell lines. Results showed that the correlation was highly significant (*r* = 0.78, *P* = 0.007). **D.** Effect of ectopic expression of TXNRD1 on auranofin's activity. Calu3 cells were transfected with pcDNA3 or pTXNRD1 for 24 h and then treated with auranofin for 72 h. Cell viability was determined in a triplicate assay. The viability of control cells was set as 1. * indicating *P* < 0.05. **E.** Western blot for TXNRD1 expression in the cells described in (D).

Because of high affinity of auranofin to thiols, TXNRD, the only enzymes that catalyze the reduction of thioredoxin (TXN) with electrons from NADPH [27, 28], have been identified the major targets of the gold-containing drugs such as auronofin [19, 29]. To determine whether auranofin's activity is associated with endogenous TXNRD1 expressions, we analyzed the TXNRD1 levels in NSCLC cell lines used in this study. Western blot analysis revealed that lung cancer cells that were highly sensitive to auranofin all expressed low levels of TXNRD1, whereas lung cancer cells with high TXNRD1 levels were relatively resistant to auranofin (Figure 3A). Enzymatic analysis revealed that cell lines with high TXNRD1 protein levels also had high TXNRD1 enzymatic activity. Correlation analysis revealed that auranofin IC_50_ values were significantly correlated with TXNRD enzymatic activity levels in lung cancer cell lines (*r* = 0.78, *P* = 0.007), demonstrating that endogenous TXNRD1 levels in tumor cells are inversely associated with auranofin's activity (Figure 3B, 3C). This results indicate that TXNRD1 expression in tumor cells may be used as a predictive biomarker to identify those most likely to respond to auranofin.

To further determine whether intracellular TXNRD1 is causally associated with the resistance to auranofin's anticancer activity in NSCLC cells, we transfected Calu3 cells with a plasmid expressing TXNRD1 or the control plasmid pcDNA3. Cells were treated with auranofin at 24h after the transfection. Cell viability was then determined at 72 h after auranofin treatment. The result showed that transient transfection of the TXNRD1 expressing plasmid rendered cells partially resistant to auranofin (Figure 3D, 3E), suggesting that high level of TXNRD1 is one of causal factors of resistance to auranofin in NSCLC cells.

### Auranofin inhibits multiple key nodes in the PI3K/AKT/mTOR pathway

**Figure 4:**
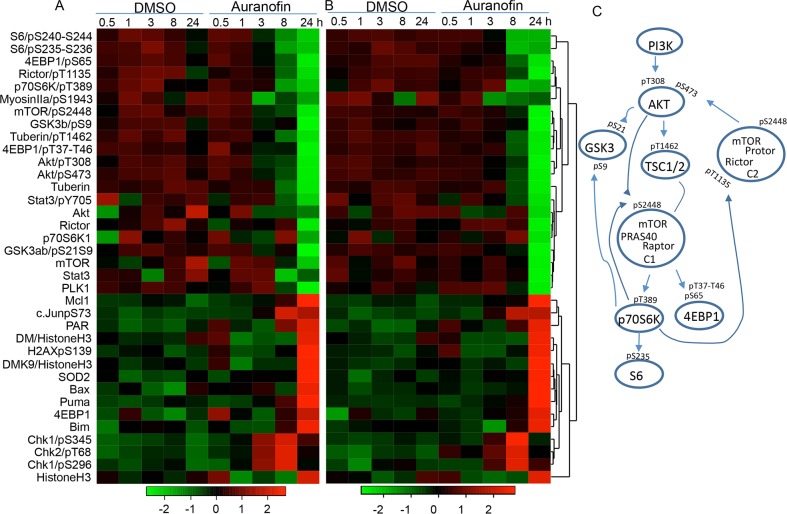
Proteomic analysis of auranofin-induced changes in proteins and protein phosphorylation in lung cancer cells Calu3 and HCC366 cells were treated with 0.5μM auranofin for the indicated times. Cells treated with DMSO were used as controls. The cell lysates were subjected to RPPA analysis of 214 protein biomarkers. **A and B.** Heatmap of the top 36 proteins changed in Calu3 (A) and HCC366 (B) cells over time. **C.** Signaling transduction in the PI3K/AKT/mTOR pathway and phosphorylations inhibited by treatment with auranofin.

To determine the mechanisms of auranofin-mediated anti-lung cancer activity, we analyzed the auranofin-induced changes in proteins and protein phosphorylation in Calu3 and HCC366 cells using RPPA, as we previously reported [30, 31]. Cell lysates were harvested after being treated with DMSO or 0.5μM auranofin for 0.5, 1, 3, 8, or 24 hours and subjected to RPPA analysis of 214 proteins or protein phosphorylation using validated antibodies available in our Proteomic Core facility. Results showed that treatment with auranofin led to drastic time-dependent suppression of several key nodes in the PI3K/AKT/mTOR pathway and in the protein translation machinery in both Calu3 and HCC366 cells, including S6, 4EBP1, Rictor, p70S6K, mTOR, TSC, AKT, and GSK3, indicating that auranofin may target multiple key nodes in the PI3K/AKT/mTOR axis (Figure 4). Most of those inhibitions occurred at 8 hours and were more profound at 24 hours. In contrast, expression of histone H3, demethylated histone H3, and pro-apoptotic proteins, such as Puma, Bax, and Bim, was dramatically induced at 24 hours, consistent with the cell death and apoptosis levels detected by annexin V and propidium iodide staining (see above). Phospho-CHEK1 and phospho-CHEK2 levels were increased at 3 and 8 hours, whereas SOD2 was increased at 24 hours, suggesting the presence of DNA damage and oxidative stress. Ingenuity Pathway Analysis (IPA) revealed that the PI3K/AKT/mTOR pathway was significantly inhibited at the time points of 8 and 24 hours (*P* = 7.5 × 10^−40^). The top upstream regulators identified by IPA are AKT, PTEN, TP53, doxorubicin and sirolimus (rapamycin) (P ≤ 1.3 × 10^−41^). This result strongly indicates that auranofin targets multiple key regulators in the PI3K/AKT/mTOR pathway and induced biological effects mimicking sirolimus. The significance of doxorubicin identified by IPA is not yet clear. However, identification of TP53 and doxorubicin in the pathway analysis may indicate that auranofin might induce DNA damage as well.

**Figure 5:**
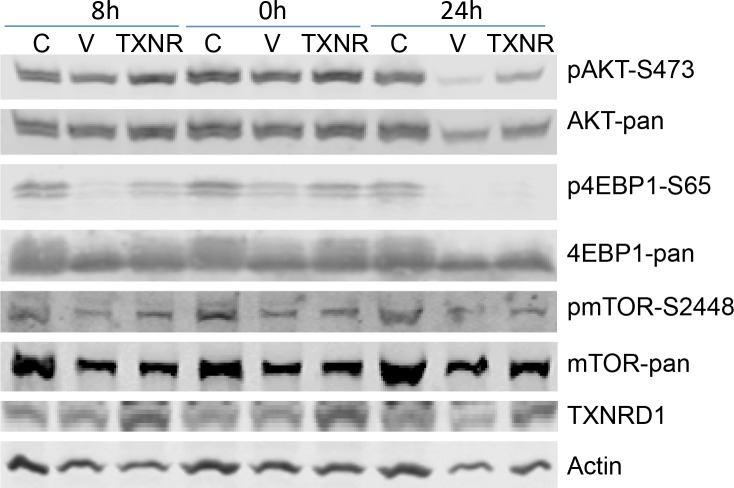
Effect of TXNRD1 expression in auranofin medicated inhibition of PI3K/AKT/mTOR pathway Calu3 cells were transfected with TXNRD1 (TXNR) or GFP (V) expressing plasmids for 24 h. Cells were then treated with 0.5 μM auranofin for 0, 8 and 24 h. Calu3 cells treated with DMSO (C) were used as controls. Pan- and phosphorylated proteins were detected by Western blot analysis. Treatment with auranofin resulted inhibition of phospho-AKT, -4EBP1, and —mTOR in vector transfected cells, which is partially reversed in TXNRD1 transfected cells.

Because transient transfection of TXNRD1 expressing plasmid in Calu3 cells resulted in partial resistance to auranofin, we analyzed whether ectopic expression of TXNRD1 has any effects on auranofin-mediated inhibition of PI3K/AKT/mTOR pathway. For this purpose, Calu3 cells were transfected with TXNRD1 or a vector control plasmid expressing green fluorescent protein (GFP). Twenty-four hours after the transfection, cells were treated with 0.5 μM auranofin. Cell lysates were harvested at 0, 8 and 24h after auranofin treatment. Calu3 cells treated with DMSO were used as controls. Phosphorylations of AKT (S473), 4EBP1 (S65) and mTOR (S2448) were determined by Western blot analysis. The results showed that ectopic expression of TXNRD1 partially reversed auranofin induced inhibition of phosphorylation of AKT, 4EBP1 and mTOR (Figure 5), indicating that TXNRD1 may participate in the regulation of PI3K/AKT/mTOR pathway and that high TXNRD1 levels can attenuate auranofin-induced inhibition of PI3K/AKT/mTOR pathway.

### Auranofin has potent *in vivo* activity against lung cancer

**Figure 6:**
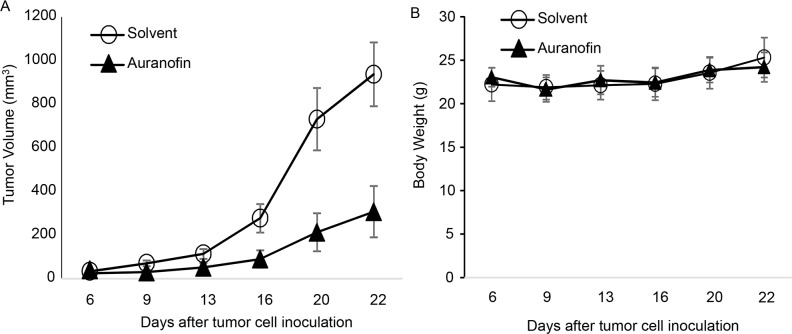
Auranofin-mediated *in vivo* activity in Calu3 xenograft tumors **A.** Tumor Volume and **B.** Body Weight of animals treated with solvent or auranofin (10 mg/kg per day). The values are mean ± standard deviation (n = 6 per group). The tumor volume in auranofin treated group is significantly different from solvent group (*P*=0.0002) when determined by ANOVA with repeated measurement module.

To determine whether auranofin had single-agent activity *in vivo* in NSCLC, we tested the activity of auranofin in a NSCLC xenograft tumor model derived from Calu3 cells. For this model, 3 × 10^6^ Calu3 cells were inoculated subcutaneously into the dorsal flank of nude mice. After the tumors grew to 3-5 mm in diameter, the mice were grouped randomly into 2 groups (n = 6) and given daily intraperitoneal solvent (2% DMSO, 8.5% ethanol, and 5% PEG-400) or auranofin (10 mg/kg) dissolved in the solvent. Tumor volume was monitored and calculated using the formula a × b^2^ × 0.5, where a represents the largest diameter and b represents the smallest diameter. Results showed that treatment with auranofin resulted in significant growth suppression of Calu3 tumors *in vivo* (Figure 6). Treatment with auranofin led to 67% inhibition of tumor growth compared with control. No weight loss was detected in any of the treatment groups, suggesting that treatment with auranofin is effective for Calu3 tumors and is well tolerated.

## DISCUSSION

Our results revealed that auranofin has *in vitro* and *in vivo* single agent activity against a subset of lung cancer cells. Auranofin alone can effectively inhibit multiple key nodes in PI3K/AKT/mTOR signaling pathways and elicit cytotoxic effects in NSCLC cells. Moreover, auranofin's anticancer activity might reversely correlate with TXNRD levels in cancer cells.

The use of auranofin for the treatment of various cancers has recently been explored [18–20], and auranofin is currently in clinical trials for the treatment of leukemia [21]. Previous mechanistic characterization has revealed that auranofin inhibits thioredoxin reductase (TXNRD) [19, 29], STAT3 [32], NF-κB [32], inflammasome receptor NLRP3 [33], proteasomal deubiquitinase [34], and selenoprotein synthesis [35]. Interestingly, NLRP3 activation mutations are present in 16% of lung adenocarcinomas and are significantly enriched for NF-κB activity [36]. Thus, most targets of auranofin are critically involved in cancer initiation, progression, and resistance to anticancer therapy. Nevertheless, using unbiased proteomic analysis, we found that treatment of lung cancer cells with auranofin inhibited phosphorylation of most key nodes in the PI3K/AKT/mTOR pathway, including S6, 4EBP1, Rictor, p70S6K, mTOR, TSC2, AKT, and GSK3, indicating that auranofin may target multiple sites in the PI3K/AKT/mTOR axis. Thus, inhibition of entire PI3K/AKT/mTOR pathway by auranofin could be a novel mechanism of action in auranofin-mediated anti-lung cancer therapy.

The PI3K/AKT/mTOR pathway is one of the major signaling pathways that regulate cell growth, proliferation, metabolism, and survival and is one of the most commonly deregulated pathways in cancer [37]. Inhibitors of the PI3K/AKT/mTOR pathway have been intensively investigated for anticancer therapy. In fact, the mTOR inhibitors everolimus and temsirolimus have been approved for the treatment of renal cancer [38] and breast cancer [39]. Nevertheless, inhibiting mTOR alone is known to activate PI3K/AKT via feedback loops [40], leading to attenuated therapeutic efficacy. Similarly, inhibiting PI3K or AKT alone resulted in feedback activation of upstream receptor tyrosine kinases [41, 42], highlighting the necessity of simultaneous targeting of multiple key nodes in cancer signaling networks for anticancer therapy to be effective. Consequently, several dual inhibitors of PI3K and mTOR, such as BEZ2359 (dactolisib) [43], PF04691502 [44], PKI-587 (gedatolisib) [45], DGC-0980 (apitolisib) [46], and GSK2126458 [47], are under extensive preclinical and clinical evaluations for anticancer therapy. As an FDA-approved drug with documented pharmacokinetic and safety profiles in humans, and having potent inhibitory effects in multiple sites of the PI3K/ATK/mTOR axis in human lung cancer cells, auranofin is likely for rapid clinical translation of its new application for lung cancer therapy.

Our results also suggested that endogenous levels of TXNRD1 expression in cancer cells are inversely associated with auranofin's activity, and causally associated with the resistance to auranofin in NSCLC cells. TXNRD/TXN is one of the major cellular enzyme systems executing an anti-oxidative stress function. Whether cells under high oxidative stress might be more vulnerable to auranofin, or whether levels of other redox regulatory systems in the cancer cells may affect auranofin's activity remains to be determined. Nevertheless, TXNRD/TXN is known to play important roles in cancer progression and anticancer therapy. TXN functions as an electron donor for ribonucleotide reductase which is critically involved in DNA synthesis [48]. The TXNRD/TXN system also catalyzes the reversible reduction of disulfides [49–51] or *S*-nitrosation [52, 53] of many cancer-associated transcriptional factors (p53, NF-kB, HIF1a), phosphatases (PTEN), kinases, apoptosis regulators (caspase-3, ASK1), and immune system modulators [54], thereby modulating the functions of the target proteins and regulating cellular redox homeostasis, DNA synthesis and repair, cell growth and survival, inflammatory response, and malignant progressions. Although little is known about the interactions between TXNRD/TXN and PI3K/AKT/mTOR pathways, our results indicate that TXNRD/TXN system may participate in the regulation of PI3K/AKT/mTOR pathway and that high TXNRD1 levels can attenuate auranofin-induced PI3K/AKT/mTOR inhibition. The association between auranofin's anticancer activity and cellular TXNRD level indicates that TXNRD expression in cancer tissue might be a predictive biomarker for identifying responders of auranofin therapy, which may facilitate patient stratification in future design of clinical trials with auranofin.

## MATERIALS AND METHODS

### Cell lines and cell culture

Human non—small cell lung cancer (NSCLC) cell lines were maintained in our laboratories as previously described [9, 55]. The authentication for each cell line was performed by DNA fingerprint analysis within 12 month. The cells were cultured in RPMI 1640 or high-glucose Dulbecco modified Eagle medium supplemented with 10% fetal bovine serum, 100 μg/mL ampicillin, and 0.1 mg/mL streptomycin; they were maintained at 37°C in a humidified atmosphere containing 5% CO_2_ and 95% air.

### Cell viability assay

Auranofin is obtained from Sigma-Aldrich Corporation. The inhibitory effects on cell growth were determined by using the sulforhodamine B assay, as described previously [56, 57]. Each experiment was performed in quadruplicate and repeated at least three times. The relative cell viability (%) was calculated using the equation OD_T_/OD_C_ × 100% (where OD_T_ represents the absorbance of the treatment group and OD_C_ represents the absorbance of the control group). The median inhibitory concentration (IC_50_) values were determined by using CurveExpert 1.3 software.

### Western blot analysis

Western blot analysis was performed as described [57]. Antibody for pan- or phospho-MLKL, AKT, mTOR, and 4EBP1, and TXNRD1 were obtained from Abcam (Cambridge MA), Cell Signaling Technology (Danvers, MA) or R&D Systems (Minneapolis, MN). Whole-cell lysates were prepared by washing the cells with phosphate-buffered saline solution (PBS) and subjecting them to lysis with RIPA buffer supplemented with the protease inhibitor cocktail. After the lysates were sonicated for 15 s, the protein concentrations were quantified using the Bio-Rad protein assay kit. Equivalent amounts of each protein were loaded, separated by 10% or 12% sodium dodecyl sulfate-polyacrylamide gel electrophoresis, and then transferred to polyvinylidene fluoride membranes at 80 V for 2 h. The membranes were blocked for 1 h with 5% nonfat dried milk in PBS buffer containing 0.1% Tween-20 (PBST) and probed with diluted primary antibody at 4°C overnight. The membranes were then washed three times in the PBST buffer and probed with infrared dye-labeled secondary antibodies. The immunoreactive bands were visualized with the Odyssey Imager (Li-COR Biosciences, Lincoln, NE).

### Biochemical and flow cytometric assays

Plasmid expressing TXNRD1 was obtained from GE Dharmacon Life Sciences (Lafayette, CO). Plasmid transfection was performed with Fugene 6 (Promega, Madison, WI), following manufactory's instruction. TXNRD1 activity assay was determined by using the TXNRD1 activity assay kit obtained from Sigma-Aldrich corporation, following the manufacturer's instructions as we previously described [11]. Auranofin-induced cytoxic effects was determined by fluorescence-activated cell sorting after staining with annexin V/propidium iodide.

### Reverse Phase Protein Array (RPPA) analysis

RPPA assay was performed at the Functional Proteomics Reverse Phase Protein Array Core facility at our institution as we previously described[30, 31]. Briefly, cells were treated with either auranofin or DMSO and harvested at the time points as indicated. Cells were then lysed in RPPA lysis buffer [1% Triton X-100, 50 mmol/L HEPES (pH 7.4), 150 mmol/L NaCl, 1.5 mmol/L MgCl_2_, 1 mmol/L EGTA, 100 mmol/L NaF, 10 mmol/L NaPPi, 10% glycerol, 1 mmol/L Na_3_VO_4_, 1 mmol/L phenylmethylsulfonyl fluoride (PMSF), and aprotinin 10 μg/mL] for 30 min with frequent vortexing on ice. The resultant solution was centrifuged for 15 min at 14,000 rpm, the supernatant was collected, and the protein concentration was determined by routine (*e.g.*, Bradford) assays and then adjusted to 1-1.5 mg/ml by lysis buffer. The samples were then submitted to the Functional Proteomics Reverse-Phase Protein Array Core facility at our institution for analysis with 197 validated antibodies specific for proteins or their phosphorylated sites that are involved in various signaling pathways. Signals from each dilution were fitted with the non-parametric model developed by the Department of Bioinformatics and Computational Biology at MD Anderson [58]. The protein concentrations of each set of slides were then normalized and corrected across samples by the linear expression values, using the median expression levels of all antibody experiments to calculate a loading correction factor for each sample, as previously described [30, 31]. Heatmap is constructed by the R gplots program.

### Animal experiments

Animal experiments were carried out in accordance with *Guidelines for the Care and Use of Laboratory Animals* (NIH publication number 85-23) and the institutional guidelines of M. D. Anderson Cancer Center. Subcutaneous tumors were established in 6- to 8-week-old female nude mice (Charles River Laboratories Inc., Wilmington, MD) by inoculation of 2 × 10^6^ Calu3 cells into the dorsal flank of each mouse. After the tumors grew to 3-5 mm in diameter, the mice were grouped randomly into two groups and treated with intraperitoneal administration of 1) auranofin (10 mg/kg/day) and 2) solvent (2% DMSO, 10% ethanol and 5% polyethylene glycol 400). Tumor growth and animal body weight were monitored overtime. Tumor volumes were calculated by using the formula a × b^2^ × 0.5, where a and b represented the larger and smaller diameters, respectively. Mice werekilled when the tumors grew to 15 mm in diameter.

### Statistical analysis

Each experiment or assay was performed at least two times, and representative examples are shown. Data are reported as mean ± standard deviation (SD). Statistical significance of the differences between treated samples was determined by using the two-tailed Student *t* test. Pearson correlation (assuming normality) were used to assess whether there were associations between auranofin's anticancer activity and TXNRD1 expressions. All statistical analyses were performed using IBM SPSS program (Version 22). Differences were considered statistically significant at *P* < 0.05.
